# Spatial microheterogeneity in the valence band of mixed halide hybrid perovskite materials

**DOI:** 10.1039/d2sc03440a

**Published:** 2022-07-19

**Authors:** Axel Erbing, Bertrand Philippe, Byung-wook Park, Ute B. Cappel, Håkan Rensmo, Michael Odelius

**Affiliations:** Department of Physics, Stockholm University, AlbaNova University Center SE-106 91 Stockholm Sweden odelius@fysik.su.se +46 8 5537 8601 +46 8 5537 8713; Department of Physics and Astronomy, Uppsala University Box 516 SE-751 20 Uppsala Sweden hakan.rensmo@physics.uu.se; Department of Energy and Chemical Engineering, Ulsan National Institute of Science and Technology 50 UNIST-gil, Eonyang-eup, Ulju-gun Ulsan 44919 Korea; Division of Applied Physical Chemistry, Department of Chemistry, KTH Royal Institute of Technology SE-100 44 Stockholm Sweden

## Abstract

The valence band of lead halide hybrid perovskites with a mixed I/Br composition is investigated using electronic structure calculations and complementarily probed with hard X-ray photoelectron spectroscopy. In the latter, we used high photon energies giving element sensitivity to the heavy lead and halide ions and we observe distinct trends in the valence band as a function of the I : Br ratio. Through electronic structure calculations, we show that the spectral trends with overall composition can be understood in terms of variations in the local environment of neighboring halide ions. From the computational model supported by the experimental evidence, a picture of the microheterogeneity in the valence band maximum emerges. The microheterogeneity in the valence band suggests that additional charge transport mechanisms might be active in lead mixed halide hybrid perovskites, which could be described in terms of percolation pathways.

## Abstract 2

Microheterogeneity in valence band maximum of hybrid perovskites with mixed I/Br composition. Theoretical calculations show that trends in hard X-ray photoelectron spectroscopy with overall composition are related to variations in local environment.

## Graphical abstract

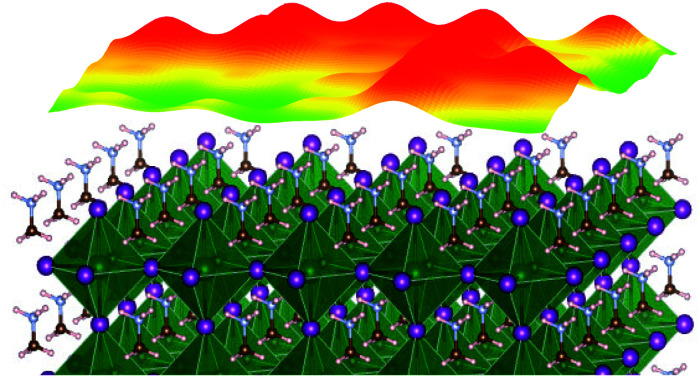

## 1 Introduction

With the current trend of shifting from traditional energy sources to renewable alternatives, solar power is becoming more prevalent and solar cells are expected to make up a significant portion of the energy production in the future.^1^ Among the most promising candidates, photovoltaics are the renewable energy resource with highest potential.^1^ Many different materials and architectures are being explored. Within a decade, the hybrid perovskite solar cell has shown overwhelming progress and has reached over 25% photon-to-power conversion efficiency (PCE),^2,3^ owing to the organic metal hybrid perovskite (OMHP) having various favorable properties such as ambipolar charge conduction^4^ and sufficiently large photon absorption range.^5^ Furthermore, it is a low-cost contender for the next-generation of photovoltaics. Recent cutting-edge technology has developed efficient tandem architecture^6–8^ and large-scale devices.^9^

**Fig. 1:**
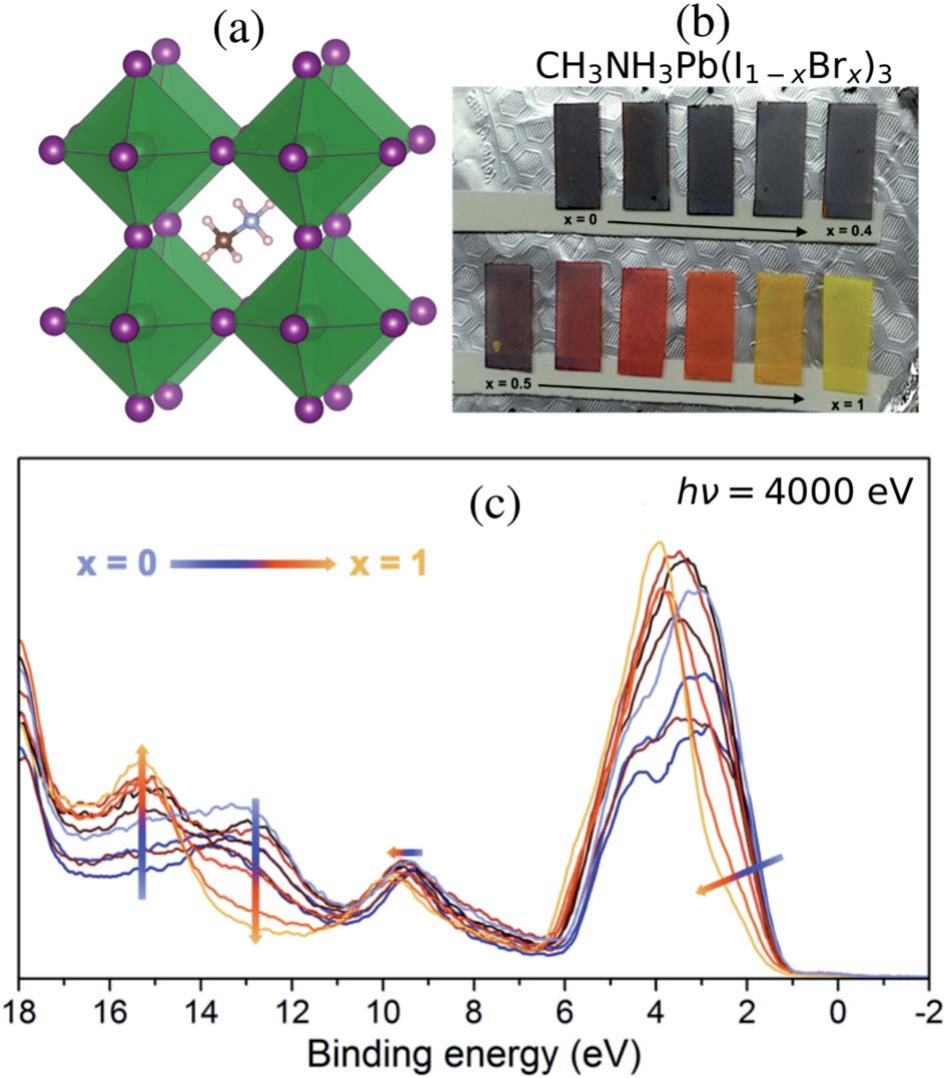
(a) The crystal structure of lead halide hybrid perovskite shows the methylammonium cation in the cavity between the inorganic octahedra with lead (green) and halide (purple) ions. (b) Samples of CH_3_NH_3_Pb(I_1−*x*_Br_*x*_)_3_ varying color with increasing Br content. (c) Valence band HAXPES measurements measured at 4000 eV for different halide compositions, displayed with a gradual change in line color from iodide dominated *x* = 0 (blue) to bromide dominated *x* = 1 (yellow).

Most OMHP materials in solar cell applications have used methylammonium (CH_3_NH_3_^+^), formamidinium and an admixture of cesium cations at the A site of the perovskite AMX_3_ structure (A: organic, M: metal di-cation and X: halide) in the cavities between corner-sharing octahedra with lead cations in the center M site. The methylammonium lead tri-iodide (CH_3_NH_3_PbI_3_) system^10–12^ in Fig. 1(a) has popularly been used in the many fundamental investigations as it is one of the standard materials. However, several material properties are still veiled. In particular, the mixed halide systems of CH_3_NH_3_PbX_3_ still need to be characterized in terms of their energy structures for understanding charge transportation processes and crystal stability. CH_3_NH_3_Pb(I_1−*x*_Br_*x*_)_3_ materials with mixed halides in the X site based on the combination of the pure CH_3_NH_3_PbI_3_ and CH_3_NH_3_PbBr_3_ crystals have been reported by Noh *et al.*^13^ Another approach has been to use a mixture involving a third halide, chloride; formally CH_3_NH_3_Pb(I_1−*x*_Br_*x*_)_3−*y*_Cl_*y*_, in the synthesis as reported by Park *et al.*^14^ allowing for accurate control of the composition between CH_3_NH_3_PbI_3_ and CH_3_NH_3_PbBr_3_ as displayed for the samples in Fig. 1(b). Notably, it has been shown that incorporating Cl in the synthesis of OMHP can contribute to retaining crystal stability for a longer period.^15,16^ Anion substitution has also been used to reduce defects in OMHP materials and at their interfaces to oxide materials which lead to improved performance.^17–19^ Mixed halogen compositions have been shown to enhance performance of light emitting devices.^20^ In theoretical studies, light-induced phase separation into Br-rich and I-rich domains has been proposed as an explanation for photoinstability.^21^

The halide ions are at the connection between the corner-sharing octahedra which results in an inorganic framework with an electronic structure that dominates the valence band maximum^10,22^ and the conduction band minimum,^23^ and determines photovoltaic characteristics of these solar cell materials. The halide ions also interact with methylammonium through hydrogen bonding as previously investigated from the point of view of the –NH_3_^+^ group with nitrogen K-edge X-ray absorption spectroscopy and electronic structure calculations.^24^ Extensive investigations of the hydrogen bonding interaction in hybrid perovskite materials have also been performed with vibrational spectroscopy and associated modelling.^25–28^ The hydrogen bonding yields a slight nitrogen admixture in the upper valence band, which recently has been discussed in the context of nitrogen K-edge X-ray emission^29^ and resonant Auger^30^ spectroscopy. With variations of the A ions in lead bromide perovskites, it has been shown in measured and simulated bromine K-edge X-ray absorption spectra that hydrogen bonding strongly influences bromine σ* and π* bands in the conduction band.^31^

In general, the halide X site can be occupied by I, Br, Cl, or in principle any combination of the three for the system of CH_3_NH_3_Pb(I_1−*x*_Br_*x*_)_3−*y*_Cl_*y*_. Variation in the halide composition has previously been demonstrated to strongly influence both the band gap in the material and the PCE of the solar cell.^32^ Since the halide ions have a dominant contribution at the top of the valence band,^10,22,33^ the exact composition can also have an influence on charge dynamics. The electron and hole mobility in hybrid perovskites is moderate, but this is compensated for by long charge recombination life times.^34^ An important mechanism for charge carrier mobility is governed by the electrostatic interactions involving phonon modes in the inorganic lattice.^34^ Intrinsically, CH_3_NH_3_PbX_3_ has a charge-carrier mobility on the same order of magnitude for both electrons and holes, more akin to semiconductors such as GaAs or Si. The substitution of I with Cl has been shown to have only a minor effect on the charge carrier mobility,^35^ and with Br there is a reduced mobility.^34^ It is important to understand how the halide composition effects the electronic properties of CH_3_NH_3_PbX_3_. In particular, combined molecular dynamics and quantum mechanical calculations on explicit structural models of mixtures can be used to identify and isolate microscopic effects of different local environments, including electronic structure and bond length. Hence, in this combined experimental and theoretical study, we are investigating the influence of halide mixtures on the electronic structure of the materials with density functional theory (DFT) and hard X-ray photoelectron spectroscopy (HAXPES) for systems with varying composition.

## 2 Materials and methods

### 2.1 Experimental details

Mixed halide perovskites were synthesized following the procedure previously reported.^14^ The ratio between the halide and lead was kept at 3 : 1 using precursors of methylammonium iodide and bromide salts (CH_3_NH_3_I, CH_3_NH_3_Br) and PbCl_2_ was used as the source for the lead ion. The final precursor solutions contained a solvent mixture of dimethylformamide, dimethylsulfoxide, and tetrahydrofuran. The substrate was fluorine-doped tin oxide (FTO) coated glass (Pilkington TEC 15) covered with a compact TiO_2_ as well as a mesoporous titanium dioxide (mp-TiO_2_) layer^14^ in the order of a few hundred nm. The perovskite precursor solutions were used to spin-coat the different perovskite compositions onto the substrates. The perovskite material completely covered the substrate with a thickness substantially larger than the probe depth of the HAXPES measurements and thus there are no effects from TiO_2_ substrate in the reported spectra. Although the precursors contain chloride ions, previous investigations have shown that the organic–inorganic lead mixed halides do not contain any substantial amounts of chlorine in their final structure.^14,36^ This is particularly true in the surface region where no Cl 2p peak could be observed^37^ and where the valence structure was largely unaffected by the presence of chloride ions in the synthesis. Therefore while the effect from Cl may still have impact on the kinetics of the perovskite formation and therefore on the film morphology, we will in the following describe the films as CH_3_NH_3_Pb(I_1−*x*_Br_*x*_)_3_ and also exclude the chloride ions in the theoretical description. Varying the composition of halide ions at the X sites allows us to influence the properties of lead halide hybrid perovskites. Materials with ionic mixtures have been shown to have superior photovoltaic properties.^38^ In Fig. 1(b), we show thin-film samples of CH_3_NH_3_Pb(I_1−*x*_Br_*x*_)_3_ perovskites (from *x* = 0 to *x* = 1 in steps of 0.1) as deposited on the mp-TiO_2_ layer. In Park *et al.*,^14^ it was concluded from XRD measurements that a transition from a tetragonal to a cubic structure occurs for a Br fraction larger than 0.3. The lattice constants change gradually with the bromide fraction *x*, but inbetween *x* = 0.3 and *x* = 0.4 there is a kink, attributed to the transition from a tetragonal to a cubic structure. The electronic structure of this series of samples was probed in HAXPES measurements.

Measurement with HAXPES was performed on the prepared samples at a photon energy of 4000 eV at the GALAXIES beam line at the synchrotron SOLEIL.^39^ At this photon energy, the probing depth for valence electrons is approximately 21 nm (ref. 40) and the valence band HAXPES data therefore reflects more of the bulk of the sample than measurements with a home-lab X-ray source such as Al K_α_. The photoelectron spectra were recorded with a Scienta Omicron EW4000 HAXPES hemispherical analyzer in fixed mode using a pass energy of 500 eV. These settings were chosen to minimize the measurement time and therefore to avoid sample degradation by X-rays. Furthermore, the measurement spot was only exposed to X-rays during the measurement. The measured spectra were normalized at the peak of the Pb 5d_5/2_ core level and energy calibrated with alignment at the Fermi level of Au at zero binding energy. In the data analysis, we realized that the measured HAXPES data for the CH_3_NH_3_Pb(I_1−*x*_Br_*x*_)_3_ sample at *x* = 0.9 was suffering from large surface sample contamination, and it was discarded when reporting the series of spectra.

### 2.2 Computational details

As motivated in the experimental details above, we will theoretically investigate the electronic structure of the experimental samples in Fig. 1(b) using models of CH_3_NH_3_Pb(I_1−*x*_Br_*x*_)_3_ with varying composition. To describe the electronic structure of these mixed halide OMHP materials, we employed DFT as implemented in the CP2K software suite,^41,42^ using the Gaussian and Plane Wave (GPW) method^43^ with a Goedecker–Teter–Hutter (GTH) pseudo-potential description.^44–46^ The Kohn–Sham orbitals were expressed in Gaussian double-ζ (DZVP-GTH) basis sets (of MOLOPT type for Pb/I/Br)^47^ whereas the electron density was described with an auxiliary plane wave basis with a kinetic cut-off of 300 Ry. The calculations were performed at the Γ point using the Perdew–Burke–Ernzerhof (PBE) gradient corrected exchange correlation functional^48^ augmented with Grimme's D3 van der Waals interactions.^49^ The exclusion of Brillouin zone sampling is motivated by the size of the super cell and the fact that we primarily analyze the local electronic structure.

Five realizations were constructed to obtain sufficient statistics for each of three compositions of CH_3_NH_3_Pb(I_1−*x*_Br_*x*_)_3_ with *x* = 0.1, *x* = 0.5, and *x* = 0.9 (corresponding to 10, 50 and 90% I). The pure OMHP materials have previously been studied^22^ and crystal structures of their tetragonal phases have been reported with lattice parameters *a* = 8.85 Å, *c* = 12.64 Å for CH_3_NH_3_PbI_3_ and *a* = 8.86 Å, *c* = 11.83 Å for CH_3_NH_3_PbBr_3_.^50,51^ The mixed halide lattice parameters were interpolated from the pure lattice parameters giving the following tetragonal simulations cells:

• 8.860 Å × 8.860 Å × 11.890 Å (10% I).

• 8.856 Å × 8.856 Å × 12.140 Å (50% I).

• 8.850 Å × 8.850 Å × 12.380 Å (90% I).

It should be noticed that experimentally a slight kink in the gradual change of the cell parameters have been observed^14^ associated with a change from tetragonal to cubic phase at *x* = 0.3 to 0.4 because the cubic phase of CH_3_NH_3_PbBr_3_ is more stable at room temperature.^51^ Nonetheless, we have employed a tetragonal phase along the whole series of compositions to create a continuous variation. Based on the above cell parameters, 2 × 2 × 2 super cells containing 32 Pb^2+^, 96 X^−^, and 32 CH_3_NH_3_^+^ ions were constructed by scaling an initial structure from a previous MD simulation trajectory of CH_3_NH_3_PbI_3_.^10^ The desired ratio of I : Br was selected and these halide ions were distributed randomly at the X sites in the simulation cell. Hence, the halide ions are assumed to occupy the same lattice sites in the mixed materials as in the pure halide perovskites, which can exist in the same phase,^38^ even though the bromine material is at room temperature when single crystal. This compromise of the realism in the modelling was preferred since we wanted to make a direct comparison of different compositions. For each realization, short *ab initio* molecular dynamics simulations were performed for 2.5 ps in the canonical ensemble at 300 K to allow for relaxation of the geometries and dynamical effects in the calculation of the density of states. Two snapshots, at times *t*_1_ and *t*_2_, from the MD simulations were selected and used in calculations of the electronic density of states (DOS). The two time steps were separated by 1 ps. Single-point calculations were performed for each composition, realization and two different time snapshots, altogether 30 different configurations, in each of which we calculate the locally projected DOS (PDOS) of every lead ion and halide ion. The effect of spin–orbit coupling was neglected, since we focus on the direct influence of ion distribution and orbital mixing. The obtained PDOS data was averaged over the five realizations and over two MD snapshots. A constant ad hoc shift of 1.975 eV was applied to the theoretical data in order to match the energies of the main valence features with the experimental results. This was required due to limitations in present pure DFT approximations, in which the energy derivative discontinuity is not well-described.^52,53^ Hence, the eigenvalues of the Kohn–Sham orbitals are not strictly relatable to binding energies in the photoelectron spectra, but pragmatically they are still very useful for interpretation. Additional calculations were performed using the hybrid HSE06 (ref. 54 and 55) functional, to evaluate the sensitivity to the choice of functional and in particular the effect of inclusion of exact exchange. Analysis of the PDOS and not solely the total DOS is essential, since the photoionization cross section of different elements varies with incident photon energy. Finally, the discrete PDOS data was convoluted by a Gaussian broadening function with a full width at half maximum (FWHM) of 0.4 eV in order to directly compare with the experimental HAXPES data.

In order to investigate the effects of local halide environments on the Kohn–Sham orbitals as monitored by the PDOS, ions of the same element were classified by the halide composition of their immediate surrounding. The Pb^2+^ ions make up the centers of the corner-sharing octahedra with the halide ions on the vertices. Hence, each lead ion is surrounded by six halide ions giving rise to a total of seven different classes of local environments ranging from pure I coordination to pure Br coordination. Notice that only the halide composition in this environment, not the precise arrangement in the coordination, is taken into account in this scheme.

It is not as straightforward to classify the environment of the I^−^ and Br^−^ ions. The halide ions were not directly bonded to other halide ions but instead each occupy a shared vertex of two Pb centered octahedra. Considering both these octahedra to define the local environment, each halide anions is *via* its two octahedra connected to ten other halide anions, five on each octahedron, as next neighbors. This results in a total number of eleven different local environments. For N and C atoms in methylammonium, several classification schemes were attempted but no local dependence in their PDOS could be identified, probably related to the large reorientational flexibility of the organic cations. Hence, we will focus solely on the inorganic framework.

## 3 Results and discussion

In this study, we investigate the electronic structure in the valence band in mixed I/Br perovskite materials as probed with HAXPES of the valence band in the region 0–20 eV below the Fermi energy. Variations in the I : Br ratio cause changes in the band structure, which are reflected in the color of the materials seen in Fig. 1(b). The band gap increases with the amount of Br as measured by the *x* value in the composition of CH_3_NH_3_Pb(I_1−*x*_Br_*x*_)_3_ samples, and the absorption is blue-shifted in the visible spectrum, giving the samples a black color for low *x* gradually converting through red for *x* = 0.7 and finally becoming yellow for *x* = 1. Spectral changes in the HAXPES data with composition are displayed in Fig. 1(c). We observe an increasing binding energy and a narrowing in the shape of the main peak at 1–6 eV with increasing *x*. The peak is dominated by the I 5p and Br 4p levels, where Br 4p has a higher binding energy than I 5p – a result of an intrinsic difference between this pair of halide ions. These levels also involve mixing with Pb 6s and Pb 6p.^33^ At low *x*, the peak is broad both due to a noticeable spin–orbit coupling^56^ in I 5p and a larger sensitivity to fluctuations in bond length. The changes in the electronic structure can be understood from the atomic electronic differences in the I^−^ and Br^−^ ions reflected in the pure materials,^10,22^ which has also been investigated for more complex materials (including mixtures at both the A cation and X anion sites) by scanning the incident energy in photoelectron spectroscopy.^33^ We also notice the gradual increase in the peak of the Br 4s levels at 15 eV and decrease of the peak of I 5s levels at 13 eV, which are mixing with the Pb 6s and Pb 6p.^33^ Finally, there is a small spectral response in the Pb 6s dominated feature at 9–10 eV with an increasing binding energy for increasing *x*, clearly showing how the halide ions influence the electronic structure around lead.

**Fig. 2:**
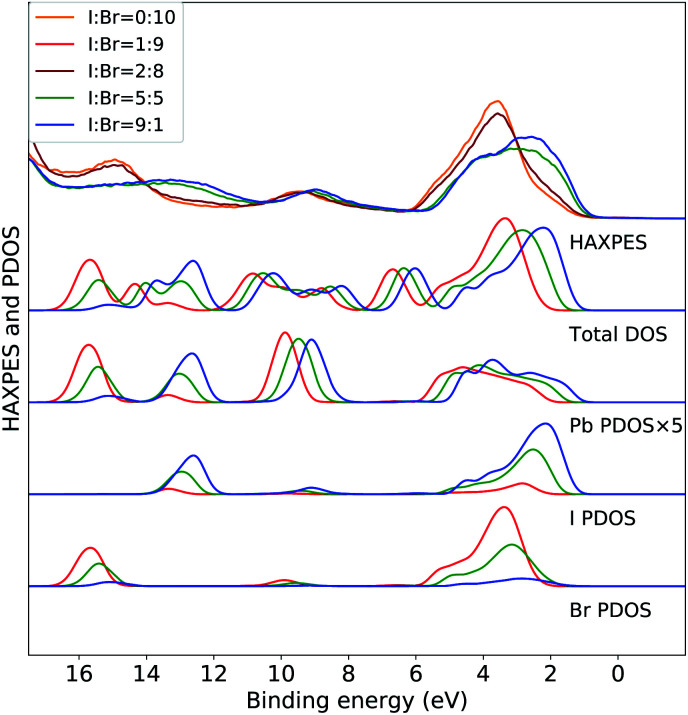
Valence band HAXPES measurements of CH_3_NH_3_Pb(I_1−*x*_Br_*x*_)_3_ samples for different halide compositions from Fig. 1 compared to total DOS and PDOS of lead and halide ions from DFT calculations on the CH_3_NH_3_Pb(I_1−*x*_Br_*x*_)_3_ models. Different overall compositions are distinguished by line colors: blue *x* = 0.1 (90% I), green *x* = 0.5 (50% I), and red *x* = 0.9 (10% I). Notice that the experimental data for *x* = 0.9 was suffering from large surface sample contamination, and we replaced it with experimental spectra for *x* = 0.8 and *x* = 1.0, being very similar. All calculated PDOS curves are shifted ad hoc to align with the main experimental feature around 3 eV. The same shift (see Methods section) is used for all compositions. Noticed that the PDOS of lead has been enhanced for clarity.

The variations in the experimental HAXPES spectra in the Fig. 1(c) are further analyzed in DFT calculations on CH_3_NH_3_Pb(I_1−*x*_Br_*x*_)_3_ models for composition with 10, 50 and 90% I. In Fig. 2, the trend in the projected density of states in the models for different compositions are compared to the corresponding experimental HAXPES data from Fig. 1(c). For *x* = 0.1 and *x* = 0.5, we can compare experimental and theoretical data directly, but the measurement for *x* = 0.9 had to be discarded. Hence, the measurements for the CH_3_NH_3_Pb(I_1−*x*_Br_*x*_)_3_ sample with *x* = 0.8 as well as the pure Br sample are included for comparison to the theoretical results from the 10% I composition. By separately displaying the PDOS for the three inorganic ions, dominating the HAXPES spectra at 4000 eV incident energy, we can follow see how the experimental spectra are built up from contributions of lead, bromine and iodine. For clarity, the intensity of the Pb PDOS is increased by a factor of five.

**Fig. 3:**
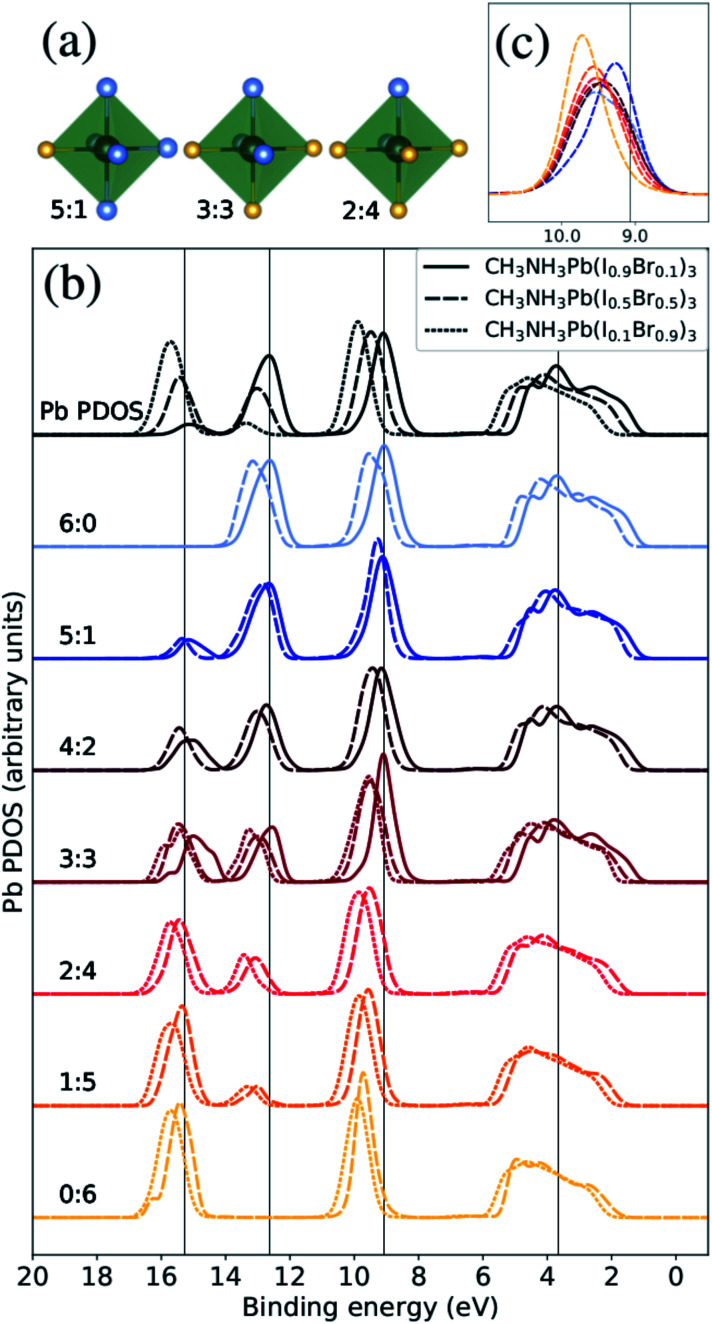
The Pb PDOS is decomposed into contributions from different classes of local coordination and local electronic structure of lead ions. The analysis is performed for theoretical CH_3_NH_3_Pb(I_1−*x*_Br_*x*_)_3_ models with *x* = 0.1 (solid lines), *x* = 0.5 (dashed lines), and *x* = 0.9 (dotted lines). The same energy shift (see Methods section) as in Fig. 2 was applied. (a) Examples of three classes of the Pb atoms. The iodide atoms as blue and the bromine atoms as yellow. (b) Local density of states for different local environments for Pb. (c) Highlighted shifts in the peak at 9–10 eV for varying local environments at *x* = 0.5 composition.

**Fig. 4:**
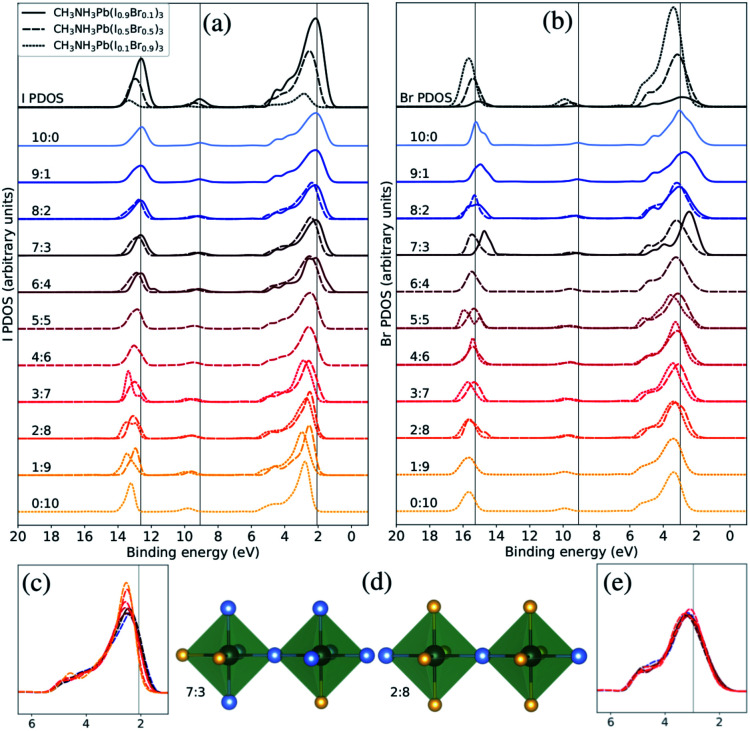
Both halide PDOS are decomposed into contributions from different classes of local coordination and local electronic structure of halide ions. The analysis is performed for the theoretical CH_3_NH_3_Pb(I_1−*x*_Br_*x*_)_3_ models with *x* = 0.1 (solid lines), *x* = 0.5 (dashed lines), and *x* = 0.9 (dotted lines). The same energy shift (see Methods section) as in Fig. 2 was applied. (a) Local density of states for different local environments for I. (b) Local density of states for different local environments for Br. (c) Highlighted shifts in the peak at 3 eV for I in the *x* = 0.5 composition. (d) Examples of two classes of local environment around the halide ions. The iodide atoms as blue and the bromine atoms as yellow. (e) Highlighted shifts in the peak at 3 eV for Br in the *x* = 0.5 composition.

The calculations of the total DOS and the experimental HAXPES data are in qualitative agreement with the exception of the contributions associated with the methylammonium (CH_3_NH_3_^+^) counter ion, for instance at 6, 8, and 12 eV. These discrepancies can be attributed to low photoionization cross section for carbon and nitrogen at the employed incident X-ray energy. We observe an obvious gradual increase in Br PDOS at the expense of I PDOS as a function of I : Br ratio and hence with increasing *x*, both in the I 5p/Br 4p peak at 0–6 eV and the peaks at 13 eV from I 5s and 16 eV from Br 4s, clearly reproducing the experimental trends. In the region of the valence band maximum (0–6 eV), at first approximation the relative intensity of the iodide and bromide contributions gives an apparent shift of the bands in the experimental feature. This leads to the important conclusion, that in all halide hybrid perovskite materials with mixed I/Br halide composition, the very top of the valence band consists of I 6p states but with negligible bromide contribution. In agreement with earlier studies,^10,33^ these levels are hybridized with lead in Pb 6s–I 5p anti-bonding levels. The observed shift of approximately 1 eV is reproduced but slightly overestimated by the calculations. Similarly, the shift towards higher binding energies of the smaller Pb 6s peak at 9 eV is also captured in the theoretical models. In addition to this, we also identify an additional effects of the composition. We observe that the PDOS of each element, systematically shifts to higher binding energy with increasing *x*. This implies that the changes in the relative amount of Br^−^*versus* I^−^ ions influences the electronic structure at the non-local level. This is possibly an artifact in the periodic DFT calculations, since the orbital energies in the different systems are not strictly comparable, and below we will study primarily variations within each model. The I 5s and Br 4s peaks at 13 eV and 16 eV, respectively, involve mixing predominantly with Pb 6s. In Fig. 3 and 4, we further analyze the effects of mixing iodide and bromide in the lead halide hybrid perovskite, by decomposition of the average PDOS of each element into contributions from different local environments.

A key to gain a deeper understanding of the trends in the experimental HAXPES spectrum in Fig. 1(c) is to study the variations in local electronic structure at a fixed composition, as an alternative to calculate the average electronic structure at varying composition. The exact distribution of different halide ions at different mixed compositions is difficult to establish experimentally, and the observables anyhow correspond to averaged properties. In the theoretical models however, where the halide ions are distributed randomly according to the desired I : Br ratio, we can characterize the local coordination in great detail. This means that we can investigate how the local electronic structure depends on the local coordination. We will again use the tool of PDOS to sample the local projection of the density of the Kohn–Sham orbitals. Using the CH_3_NH_3_Pb(I_1−*x*_Br_*x*_)_3_ models analyzed in Fig. 2 for 10, 50 and 90% I composition, we have investigated different possible local environments, as discussed above.

In silver bismuth halide perovskites, we investigated the energetics using Monte Carlo simulations^57^ of the distribution of ions and valencies searching for optimal configurations, but in the current study we seek to investigate a broad distribution of different local environments and therefore we use a randomized procedure for distributing the halide ions over the X sites. To begin with, we will discuss the coordination around the Pb^2+^ ions and its effect on the local electronic structure. Following the above discussion, we denoted these coordination classes by N_I_ : N_Br_ where for example a Pb^2+^ ion with only a single I^−^ neighbor belongs to the 1 : 5 class and a Pb^2+^ with four I^−^ neighbors falls into the 4 : 2 class. In Fig. 3(a), we display three examples of the seven possible local environments (ranging from 6 : 0 to 0 : 6 in I : Br) around each lead site classified simply in terms of the number of I^−^ and Br^−^ ions coordinated. This classification scheme for Pb environments does not distinguish the exact arrangement in the first coordination shell nor the more extended structure, but as we will show it allows us to capture important variations in the electronic structure. Notice also that most of these local environments can be found in any material composition, but the frequency of occurrence depends on *x*, denoting the overall I : Br ratio. Hence, when we compare the local PDOS of different local coordination in Fig. 3(b), each spectrum is normalized with respect to the representatives (occurrence) in each class giving comparable intensities in all cases. In addition, only classes with at least three atom representatives were included in the analysis to reduce the dependence on the specific MD snapshots. This means that we can directly monitor how the Pb PDOS varies with the local coordination in the models of the three compositions. For comparison, the average Pb PDOS is presented at the top of Fig. 3(b). Notice however that because of the limited number of realizations and finite models, all environments are not present for all compositions. For example in iodine rich composition (*x* = 0.1), there are no examples of local environments with a surplus of Br around Pb (2 : 4, 1 : 5, or 0 : 6).

The most notable trend in Fig. 3(b) is the intensity variations in the peaks at 13 eV and 16 eV associate with the mixing with the I 5s and Br 4s levels, which distinctly vary with the number of iodide and bromide ions in the first coordination shell regardless of overall composition. This signifies the bonding between lead and halide ions but the levels are dominated by halides which hence determine the energy positions. This systematic variation in the computational models closely matches the trend in the HAXPES measurements on the series of samples in Fig. 1. It is noteworthy that the local electronic structure as monitored in the Pb PDOS is very similar in the different compositions, apart from the fact that iodide local environments are rare in bromine rich compositions, and the other way around. Additionally, there is a slight shift towards higher binding energies of the peak at approximately 9–10 eV. The shift in this peak is clearer for the CH_3_NH_3_Pb(I_1−*x*_Br_*x*_)_3_ model with *x* = 0.5, highlighted in Fig. 3(c), as the full range of possible local environments are represented in this case. The magnitude of this is 0.45 eV, smaller than the shift in the total Pb PDOS between CH_3_NH_3_Pb(I_1−*x*_Br_*x*_)_3_ at *x* = 0.1 and CH_3_NH_3_Pb(I_1−*x*_Br_*x*_)_3_ at *x* = 0.9 but more in line with the HAXPES measurements. Finally, we notice an general shift related to the overall composition and little changes in peak shapes. We conclude that the local electronic structure at the lead ions, as probed in the Pb PDOS, exhibits trends with changes in coordination that mimic those associated with global composition. Hence, the contribution of lead in the valence band is determine by the orbital mixing with the coordinating halide ions.

Next, we will change the view point and study the electronic structure of the halide ions. These exhibit a weaker dependence on the local structure, since the changes only occur in the second coordination shell, indirectly through the coordination of halide ions around the lead ions. There are ten halide ions in the different local environments around the X sites. In Fig. 4(d), we display two of the eleven possible local environments ranging from 10 : 0 to 0 : 10 in I : Br in a classification which again neglects variation in the exact arrangement of the ten neighboring halide ions (two Pb–X bonds away). The I PDOS and Br PDOS for these classes of local environments are displayed in Fig. 4(a) and (b), respectively. Since, the first coordination shell containing two lead ions is preserved in all the halide classes, the changes in the local electronic structure around the halide ions are more subtle than in the case of the lead ions. However, several important changes can be identified. Looking carefully at the binding energy of the features in the I PDOS in Fig. 4(b), we observe a similar shift in the I 5s peak as in the lead (Pb 6s)-dominated peak at 9–10 eV for Pb PDOS seen in Fig. 3(b). We also analyzed the very weak features for the halides at binding energy of 9–10 eV corresponding Pb 6s and can conclude that they follow the same trend. In addition, in the I PDOS, a significant narrowing of the peak with increased Br content is observed, most apparent for the CH_3_NH_3_Pb(I_1−*x*_Br_*x*_)_3_ model at *x* = 0.5 composition highlighted in Fig. 4(c), for increasing fraction of bromide ions in the second coordination shell of iodide. This indicates that the mixing of the iodide orbitals stretches over the directly coordinated lead ions. The narrowing of the upper valence band has also been observed in previous comparison of the pure materials.^22^ For Br, no clear environment-dependent trend could be observed in this feature in Fig. 4(b) or in the highlighted region in Fig. 4(e). The small irregularities in these peaks are likely due to poor statistics for those particular combinations of class and composition. Notice in particular, that these trends can be seen for different local environment in the same composition, but are also reflected in the averaged PDOS for each element and its variation with overall composition as seen in Fig. 2. Despite that the overlap is mediated through the bonding with the lead ions, the cooperative effect in orbital mixing between next-neighbor halide ions results in the trend that the binding energy of the local halide PDOS decrease with increasing number of next-neighbor iodide ions. Through the same mechanism, the dispersion of the band at the top of the valence increases. We conclude that the larger and more polarizable iodide ions are more susceptible to influences from the next-neighbor interactions than the bromide ions. The analysis of local electron structure sheds light on the mechanisms for the trends with composition, and gives valuable insight into the spatial heterogeneity of the valence band.

**Fig. 5:**
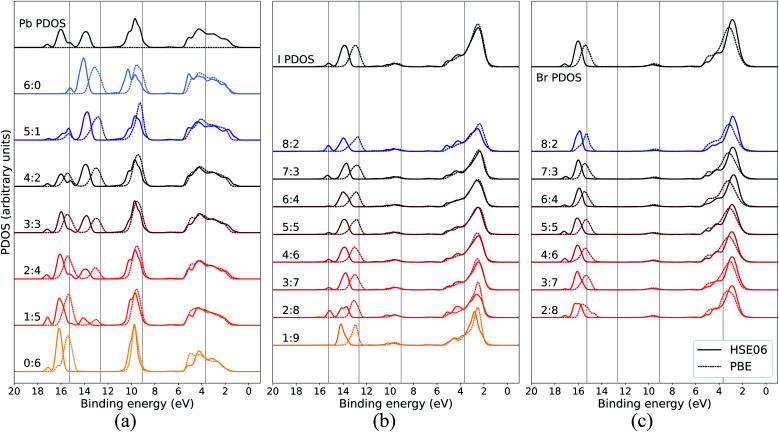
Local coordination and local electronic structure using the PBE GGA functional and the HSE06 hybrid functional, shown in dashed and solid lines respectively, of CH_3_NH_3_Pb(I_0.5_Br_0.5_)_3_ for (a) Pb, (b) I, and (c) Br. The same energy shift (see Methods section) as in Fig. 2 was used for the GGA results while the HSE06 PDOS was shifted by 1.245 eV. The PBE results for local environments at *x* = 0.5 are reproduced from Fig. 3(b), 4(a) and (b).

In order to assure that the above results remain also for higher levels of theory, additional calculations using a hybrid functional were performed for comparison. In Fig. 5, the calculated PDOS for Pb, I, and Br in the CH_3_NH_3_Pb(I_1−*x*_Br_*x*_)_3_ model at *x* = 0.5 at the GGA level is compared to the corresponding results using the hybrid HSE06 functional. The hybrid results are aligned with the previous PBE results using a constant, uniform shift of 1.245 eV. The most notable difference between the two functionals is the relative binding energy between the top of the valence, as seen in I 5p and Br 4p, and the deeper I 5s and Br 4s levels which is 1–2 eV larger for HSE06 compared to PBE. Hence, despite the general improvement in the band gap of hybrid perovskite materials with hybrid functionals,^58^ the inclusion of exact exchange does not for the current system lead to an overall improvement in reproducing the relative energies within the valence band and PBE has the closest agreement of PDOS with the experimental HAXPES data. There is additional splitting of electronic orbitals associated with the partial inclusion of exact exchange in HSE06 as seen in the peaks around 14–18 eV. This is most pronounced for atoms in iodide-rich environments, and barely observable otherwise. We also notice that for HSE06 the Pb 6s feature is not exhibiting the same trend as in the PBE calculations. Apart from the above mentioned, the PDOS from the two functional approximations exhibit the same trends in energy shifts and intensity redistribution, as identified above, showing the stability of our conclusions about the variations of the local environment mimicking the changes in overall composition.

Hybrid perovskite materials with multiple mixed ion composition have been shown to give high performance in solar cell applications.^38,59^ The high PCE can have different reasons, and in particular charge carrier mobility and electron–hole recombination life time are related to the details in the electronic structure of the materials.^34,60^ Charge-carrier dynamics in hybrid perovskite materials has been thoroughly investigated.^61–64^ Our study relates to the spatial overlap in the valence band which is an important electronic parameter for hole transport. The very top of the valence band consists of I 6p states hybridized with lead but with negligible bromide contribution. The overall trends with composition observed in the HAXPES measurements can be explained mechanistically in terms of trend in local electronic structure with local coordination at fixed composition. In particular, we could show that when iodide has an iodide in the second coordination shell, the mixing of the iodide orbitals stretches over the directly coordinated lead ion. This cooperativity creates a spatial microheterogeneity in the valence band with regions of low binding energy density of states, which can be connected into pathways depending on the I : Br ratio. We show that mixing of I : Br in lead halide hybrid perovskite creates inhomogeneity in the valence band maximum. The inhomogeneity acts as a symmetry breaking which can have similar influence as grain boundaries and defects.

These observations, based on the combination of photoelectron experiments and theoretical modeling, indicate that there is a spatial heterogeneity at the top of the valence band, which can have consequences for hole transport in hybrid perovskite materials with mixed I/Br halide composition. We also speculate that the bottom of the conduction band could show similar spatial variations, which would imply that the relative overlap between orbitals at the valence band maximum and conduction band minimum strongly influences charge recombination, if there is distinct spatial distributions of hole and electron charge carriers.

## 4 Conclusions

In conclusion, the spatial heterogeneity on an atomic length-scale at the top of the valence band, discussed in the analysis above, has implications for how to understand the spatial extend of optical excitations, charge carrier dynamics, charge separation and charge carrier recombination. We see that variations with composition are mirrored by the dependence on the local environment, and also give information about local variations at a given composition. The variation on local environments around lead in mixed halide OMHP materials is also detectable in nuclear magnetic resonance spectroscopy, where they are manifested in the chemical shift, and both their connectivity and anion mobility have been explored.^65,66^ The microheterogeneity indicates that percolation theory^67,68^ could be a suitable approach to formulate models for charge transport in hybrid perovskite materials with mixed ion composition, as suggested for other semiconductors. In contrast to the nanoscopic heterogeneity effecting charge transport in dye-sensitized solar cells,^69^ we bring attention to the electronic microheterogeneity in the crystalline hybrid perovskite material itself. Similar to that investigated in previous studies of conductivity of perovskite oxide materials,^70^ the microheterogeneity induced by ion substitution in mixed halide hybrid perovskite materials could be described in the framework of percolation theory, investigating critical concentrations for halide ion mixtures.

## Data availability

Data sets generated during the current study are available from the corresponding authors on reasonable request.

## Author contributions

AB and MO performed the calculations and theoretical analysis. BP, BwP, UC, and HR performed the experiments, and BP analyzed the experimental data. AB and MO wrote the first draft and all co-authors contributed to the writing.

## Conflicts of interest

There are no conflicts of interest to declare.

## Supplementary Material

## References

[cit1] REN21 , Renewables 2021 Global Status Report, Paris, REN21 Secretariat, 2021

[cit2] Cai B., Xing Y., Yang Z., Zhang W.-H., Qiu J. (2013). Energy Environ. Sci..

[cit3] Egger D. A., Rappe A. M., Kronik L. (2016). Acc. Chem. Res..

[cit4] Biewald A., Giesbrecht N., Bein T., Docampo P., Hartschuh A., Ciesielski R. (2019). ACS Appl. Mater. Interfaces.

[cit5] Manser J. S., Christians J. A., Kamat P. V. (2016). Chem. Rev..

[cit6] Jošt M., Kegelmann L., Korte L., Albrecht S. (2020). Adv. Energy Mater..

[cit7] Forgács D., Gil-Escrig L., Pérez-Del-Rey D., Momblona C., Werner J., Niesen B., Ballif C., Sessolo M., Bolink H. J. (2017). Adv. Energy Mater..

[cit8] McMeekin D. P., Sadoughi G., Rehman W., Eperon G. E., Saliba M., Hörantner M. T., Haghighirad A., Sakai N., Korte L., Rech B., Johnston M. B., Herz L. M., Snaith H. J. (2016). Science.

[cit9] Williams S. T., Rajagopal A., Chueh C.-C., Jen A. K.-Y. (2016). J. Phys. Chem. Lett..

[cit10] Lindblad R., Bi D., Park B.-w., Oscarsson J., Gorgoi M., Siegbahn H., Odelius M., Johansson E. M. J., Rensmo H. (2014). J. Phys. Chem. Lett..

[cit11] Yoo J. J., Wieghold S., Sponseller M. C., Chua M. R., Bertram S. N., Hartono N. T. P., Tresback J. S., Hansen E. C., Correa-Baena J.-P., Bulović V., Buonassisi T., Shin S. S., Bawendi M. G. (2019). Energy Environ. Sci..

[cit12] Liu D., Yang C., Lunt R. R. (2018). Joule.

[cit13] Noh J. H., Im S. H., Heo J. H., Mandal T. N., Seok S. I. (2013). Nano Lett..

[cit14] Park B.-w., Philippe B., Jain S. M., Zhang X., Edvinsson T., Rensmo H., Zietz B., Boschloo G. (2015). J. Mater. Chem. A.

[cit15] Dastidar S., Egger D. A., Tan L. Z., Cromer S. B., Dillon A. D., Liu S., Kronik L., Rappe A. M., Fafarman A. T. (2016). Nano Lett..

[cit16] Zhao Y., Zhu K. (2014). J. Am. Chem. Soc..

[cit17] Min H., Lee D. Y., Kim J., Kim G., Lee K. S., Kim J., Paik M. J., Kim Y. K., Kim K. S., Kim M. G., Shin T. J., Il Seok S. (2021). Nature.

[cit18] Jeong J., Kim M., Seo J., Lu H., Ahlawat P., Mishra A., Yang Y., Hope M. A., Eickemeyer F. T., Kim M., Yoon Y. J., Choi I. W., Darwich B. P., Choi S. J., Jo Y., Lee J. H., Walker B., Zakeeruddin S. M., Emsley L., Rothlisberger U., Hagfeldt A., Kim D. S., Grätzel M., Kim J. Y. (2021). Nature.

[cit19] Yoo J. J., Seo G., Chua M. R., Park T. G., Lu Y., Rotermund F., Kim C. S., Moon Y.-K., Jeon N. J., Correa-Baena J.-P., Bulović V., Shin S. S., Bawendi M. G., Seo J. (2021). Nature.

[cit20] Xie M., Tian J. (2022). J. Phys. Chem. Lett..

[cit21] Brivio F., Caetano C., Walsh A. (2016). J. Phys. Chem. Lett..

[cit22] Lindblad R., Jena N. K., Philippe B., Oscarsson J., Bi D., Lindblad A., Mandal S., Pal B., Sarma D. D., Karis O., Siegbahn H., Johansson E. M. J., Odelius M., Rensmo H. (2015). J. Phys. Chem. C.

[cit23] Endres J., Egger D. A., Kulbak M., Kerner R. A., Zhao L., Silver S. H., Hodes G., Rand B. P., Cahen D., Kronik L., Kahn A. (2016). J. Phys. Chem. Lett..

[cit24] Sterling C. M., Kamal C., Man G. J., Nayak P. K., Simonov K. A., Svanström S., García-Fernández A., Huthwelker T., Cappel U. B., Butorin S. M., Rensmo H., Odelius M. (2021). J. Phys. Chem. C.

[cit25] Glaser T., Müller C., Sendner M., Krekeler C., Semonin O. E., Hull T. D., Yaffe O., Owen J. S., Kowalsky W., Pucci A., Lovrinčić R. (2015). J. Phys. Chem. Lett..

[cit26] Quarti C., Grancini G., Mosconi E., Bruno P., Ball J. M., Lee M. M., Snaith H. J., Petrozza A., De Angelis F. (2014). J. Phys. Chem. Lett..

[cit27] Senno M., Tinte S. (2021). Phys. Chem. Chem. Phys..

[cit28] Lee J. H., Lee J.-H., Kong E.-H., Jang H. M. (2016). Sci. Rep..

[cit29] Wilks R. G., Erbing A., Sadoughi G., Starr D. E., Handick E., Meyer F., Benkert A., Iannuzzi M., Hauschild D., Yang W., Blum M., Weinhardt L., Heske C., Snaith H. J., Odelius M., Bär M. (2021). J. Phys. Chem. Lett..

[cit30] Man G. J., Sterling C. M., Kamal C., Simonov K. A., Svanström S., Acharya J., Johansson F. O. L., Giangrisostomi E., Ovsyannikov R., Huthwelker T., Butorin S. M., Nayak P. K., Odelius M., Rensmo H. (2021). Phys. Rev. B.

[cit31] Man G. J., Kamal C., Kalinko A., Phuyal D., Acharya J., Mukherjee S., Nayak P. K., Rensmo H., Odelius M., Butorin S. M. (2022). Nat. Commun..

[cit32] Jacobsson T. J., Correa-Baena J.-P., Pazoki M., Saliba M., Schenk K., Grätzel M., Hagfeldt A. (2016). Energy Environ. Sci..

[cit33] Philippe B., Jacobsson T. J., Correa-Baena J.-P., Jena N. K., Banerjee A., Chakraborty S., Cappel U. B., Ahuja R., Hagfeldt A., Odelius M., Rensmo H. (2017). J. Phys. Chem. C.

[cit34] Herz L. M. (2017). ACS Energy Lett..

[cit35] Motta C., El-Mellouhi F., Sanvito S. (2015). Sci. Rep..

[cit36] Unger E. L., Bowring A. R., Tassone C. J., Pool V. L., Gold-Parker A., Cheacharoen R., Stone K. H., Hoke E. T., Toney M. F., McGehee M. D. (2014). Chem. Mater..

[cit37] Philippe B., Park B.-W., Lindblad R., Oscarsson J., Ahmadi S., Johansson E. M. J., Rensmo H. (2015). Chem. Mater..

[cit38] Jacobsson T. J., Correa-Baena J.-P., Pazoki M., Saliba M., Schenk K., Grätzel M., Hagfeldt A. (2016). Energy Environ. Sci..

[cit39] Céolin D., Ablett J., Prieur D., Moreno T., Rueff J.-P., Marchenko T., Journel L., Guillemin R., Pilette B., Marin T., Simon M. (2013). J. Electron Spectrosc. Relat. Phenom..

[cit40] Tanuma S., Powell C. J., Penn D. R. (1994). Surf. Interface Anal..

[cit41] Hutter J., Iannuzzi M., Schiffmann F., VandeVondele J. (2014). Wiley Interdiscip. Rev. Comput. Mol. Sci..

[cit42] VandeVondele J., Krack M., Mohamed F., Parrinello M., Chassaing T., Hutter J. (2005). Comput. Phys. Commun..

[cit43] Lippert G., Hutter J., Parrinello M. (1997). Mol. Phys..

[cit44] Krack M. (2005). Theor. Chem. Acc..

[cit45] Hartwigsen C., Goedecker S., Hutter J. (1998). Phys. Rev. B: Condens. Matter Mater. Phys..

[cit46] Goedecker S., Teter M., Hutter J. (1996). Phys. Rev. B: Condens. Matter Mater. Phys..

[cit47] VandeVondele J., Hutter J. (2007). J. Chem. Phys..

[cit48] Perdew J., Burke K., Ernzerhof M. (1996). Phys. Rev. Lett..

[cit49] Grimme S. (2006). J. Comput. Chem..

[cit50] Stoumpos C. C., Malliakas C. D., Kanatzidis M. G. (2013). Inorg. Chem..

[cit51] Poglitsch A., Weber D. (1987). J. Chem. Phys..

[cit52] Perdew J. P. (1985). Int. J. Quantum Chem..

[cit53] Perdew J. P., Levy M. (1983). Phys. Rev. Lett..

[cit54] Heyd J., Scuseria G. E., Ernzerhof M. (2003). J. Chem. Phys..

[cit55] Krukau A. V., Vydrov O. A., Izmaylov A. F., Scuseria G. E. (2006). J. Chem. Phys..

[cit56] Even J., Pedesseau L., Jancu J.-M., Katan C. (2013). J. Phys. Chem. Lett..

[cit57] Erbing A. , Kamal C., Johansson E. M. J. and Odelius M., (unpublished work)

[cit58] Das T., Di Liberto G., Pacchioni G. (2022). J. Phys. Chem. C.

[cit59] Bi D., Tress W., Dar M. I., Gao P., Luo J., Renevier C., Schenk K., Abate A., Giordano F., Baena J.-P. C., Decoppet J.-D., Zakeeruddin S. M., Nazeeruddin M. K., Grätzel M., Hagfeldt A. (2016). Sci. Adv..

[cit60] Johnston M. B., Herz L. M. (2016). Acc. Chem. Res..

[cit61] Ponseca C. S., Tian Y., Sundström V., Scheblykin I. G. (2016). Nanotechnology.

[cit62] Khan M. T. , Almohammedi A., Kazim S. and Ahmad S., in Charge Carrier Dynamics in Perovskite Solar Cells, John Wiley & Sons, Ltd, 2021, ch. 12, pp. 389–429

[cit63] Shi J., Li Y., Li Y., Li D., Luo Y., Wu H., Meng Q. (2018). Joule.

[cit64] Xing G., Mathews N., Sun S., Lim S. S., Lam Y. M., Grätzel M., Mhaisalkar S., Sum T. C. (2013). Science.

[cit65] Kubicki D. J., Stranks S. D., Grey C. P., Emsley L. (2021). Nat. Rev. Chem..

[cit66] Karmakar A., Bhattacharya A., Sarkar D., Bernard G. M., Mar A., Michaelis V. K. (2021). Chem. Sci..

[cit67] Last B. J., Thouless D. J. (1971). Phys. Rev. Lett..

[cit68] Introduction to Percolation Theory, ed. D. Stauffer and A. Aharony, Taylor & Francis, London, 2nd edn, 1992

[cit69] Benkstein K. D., Kopidakis N., van de Lagemaat J., Frank A. J. (2003). J. Phys. Chem. B.

[cit70] Zhang C., Kim B., Park Y. (2006). Curr. Appl. Phys..

